# Patients’ perceptions and preferences regarding telemedicine for addictive disorders

**DOI:** 10.1192/j.eurpsy.2024.57

**Published:** 2024-08-27

**Authors:** S. Achab

**Affiliations:** Psychiatry, Clinical and Sociological Research Unit, Faculty of Medicine, Geneva University, Geneva, Switzerland

## Abstract

**Abstract:**

Telemedicine is an emerging treatment option having been heaviliy used during covid lockdowns, in order to maintain traetment access including for ddictive disorders.

In the present talk, we first present data published on the challmenges met at ReConnecte the treatment facility for Addictive behaviors during pandemic.

We second, present results of a survey we conducted on preferences of telemedicine use in patients and doctors in our Geneva University Hospitals.

We finaly illustrate findings by clinical cases of patients suffering form addictive behaviors and their specific meeds and preferences in terms of telemedicine (phone or Visioconsultation).

Preferences and ehealth tools elicited depended of their psychosocial profiles, their specific needs and expected benefits from online sessions of psychotherapy.

One of the ingredients of successful psychotherapy for addictive behaviors, is teh purposeful use of telemedicine as an inztegarted treatment modality.

**Disclosure of Interest:**

None Declared

